# Surface Polyphenol Coordination Drives Efficient Foliar Deposition of Pesticide Nanocarriers

**DOI:** 10.3390/nano15231775

**Published:** 2025-11-26

**Authors:** Manli Yu, Bo Cui, Lidong Cao, Qiliang Huang, Junwei Yao, Zhanghua Zeng

**Affiliations:** 1 State Key Laboratory for Biology of Plant Diseases and Insect Pests, Institute of Plant Protection, Chinese Academy of Agricultural Science, Beijing 100193, China; yumanli01@caas.cn (M.Y.);; 2 Institute of Environment and Sustainable Development in Agriculture, Chinese Academy of Agricultural Science, Beijing 100081, China; 3 State Key Laboratory of Applied Microbiology Southern China, Institute of Microbiology, Guangdong Academy of Sciences, Guangzhou 510070, China

**Keywords:** foliar affinity, tannic acid, polylactic acid, interfacial modification, abamectin-loaded nanocapsules

## Abstract

Pesticides play key roles in modern agricultural activities. Optimizing pesticide deposition is essential for maximizing utilization efficiency and minimizing unintended environmental impacts. While electrostatic, hydrogen, and covalent interactions have been extensively studied to modulate pesticide adhesion to leaf surfaces, the potential of metal coordination bonding to enhance foliar deposition remains largely unexplored. In our work, abamectin-loaded PLA nanospheres coated in tannic acid (TA) (Abam@PLA) via the metal chelating ability of polyphenols (Abam@PLA-TA) were developed to improve abamectin retention on the surfaces of leaves. The chemical properties and morphological features of Abam@PLA-TA were characterized by scanning electron microscopy (SEM), transmission electron microscopy (TEM), Fourier transform infrared spectroscopy (FT-IR), and fluorescent imaging. The foliar retention of Abam@PLA-TA demonstrated that the tannic acid coating could significantly improve the adhesion ability and deposition efficiency of pesticides for crop leaves, which was mainly attributed to the hydrogen bonds between the polyphenols of TA and the polar groups of the wax layer. Moreover, Abam@PLA-TA exhibited better photostability capacity compared to the abamectin technical concentrate, which helps to protect light-sensitive pesticides from ultraviolet (UV) decomposition. This strategy opens up a simple but powerful avenue for the design of foliage adhesive systems and a new opportunity for the efficient utilization of pesticides.

## 1. Introduction

The main function of pesticides is to protect crops from being damaged by pests and pathogens. However, achieving direct pesticide application to target organisms remains challenging, regardless of the spray method. Therefore, crops are usually regarded as indirect targets, and pesticides are sprayed onto crop leaves to create a space that is poisonous to target organisms. It needs to be pointed out that the foliar deposition of conventional pesticides on cereal crops is typically less than 40%, and large numbers of droplets fall onto the surrounding environment through drifting, rebounding, and rolling down after spraying [1,2,3], resulting in a series of environmental problems and health risks, such as serious pollution in soil and water systems, agrochemical residues in food products, and a potential threat to human health [4,5,6,7,8]. Previous studies have shown that the wax layer on the surface of crop foliage is the main cause of the inefficient deposition of a conventional pesticide, which is mainly composed of n-alkanes, esters, aldehydes, alcohols, and triterpenoids [9,10,11,12]. These hydrophobic components restrict the attachment of droplets, resulting in serious off-target loss, reducing the utilization of pesticides. Therefore, improving the deposition of droplets on crop leaves may be a feasible strategy to improve the efficient utilization of pesticides.

In recent years, research on functional pesticide-loaded systems based on advanced materials has emerged as a research hotspot in the field of efficient pesticide application. Currently, there are two principal approaches for improving the foliar adhesion of pesticides. The first approach mainly consists of the use of surfactants to reduce surface tension [13]. The second approach is primarily related to the use of polymers to enhance the viscosity of pesticides, such as polysaccharides through hydrogen bonding and electrostatic interactions [14], polyphenols through hydrogen bonding and coordination complexation [15], and adhesive proteins through mechanical anchoring and bridge connection [16]. Through surface chemistry modification, a series of pesticide-loaded systems with excellent foliar adhesion properties have been successfully developed. For instance, fipronil-loaded microcapsules with high affinity for cucumber and peanut leaves were fabricated by coating carboxymethylcellulose with ethanediamine [17]. A polydopamine-coated graphene oxide system was designed for pesticide loss control, which maintained a higher concentration of hymexazol residue than that of a hymexazol solution containing a high concentration of surfactants [18]. A lambda-cyhalothrin-loaded Pickering emulsion with a stable gel-like network structure significantly enhanced foliar wettability compared to commercial formulations, and it further showed remarkable efficacy in controlling *Pieris rapae*, even at a 40% reduced dosage [19]. Another nanoscale delivery system, a pectin-encapsulated metal–organic framework, was constructed to improve the retention of thifluzamide in rice plants based on the affinity of pectin for plant cell walls [20].

Polylactic acid (PLA) is known as an eco-friendly material with excellent biodegradability, containing aliphatic polyesters derived from biological monomers of lactic acid. Based on its superior biocompatibility, achieving controllable release and excellent deformability, hydrophobic PLA nanocarriers have been widely applied in the field of drug delivery. Therefore, PLA also has broad prospects for the application of pesticide-loaded microcapsule carriers.

**Figure 1:**
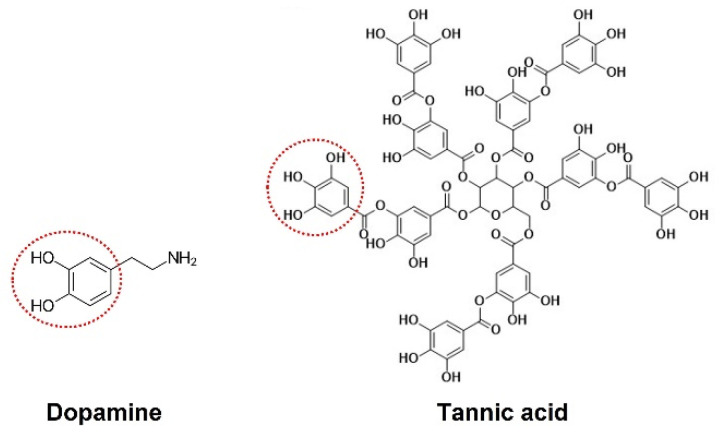
Chemical structures of dopamine and tannic acid.

Nowadays, research primarily focuses on interfacial functional modification for improving the foliar adhesion of nanopesticides [21,22]. Mussel, an ocean creature with super adhesion, can attach to nearly all materials, even in wet conditions, including the classical adhesion-resistant material poly(tetrafluoroethylene). Through systematic component analysis, it was found that the catechol groups in a dopamine molecule play a major role in this adhesive protein, which has attracted extensive attention in science [23,24,25,26]. However, due to its complex preparation process and expensive price, dopamine has lost significance in pesticide application. It is interesting that the molecular structure of tannic acid (TA) also includes large numbers of catechol groups. There are three gallic acid units on one side of the glucose core and two other gallic acid units on the opposite side (Figure 1). So it can also react with various materials through hydrogen bonds, hydrophobic interactions, electrostatic interactions, covalent bonding, and π-π stacking and is widely used as a functional material [27,28,29,30,31,32,33,34,35,36,37,38,39,40,41]. Moreover, tannic acid is a natural polyphenol and widely exists in plant roots, stems, leaves and fruit. It has some advantages in terms of being widespread, cheap, and biocompatible. However, foliar-adhesive pesticide-loaded microcapsules, which have been improved by modification with PLA with TA, have been investigated less.

In this work, abamectin, which is highly sensitive to ultraviolet (UV) light, was selected as the model pesticide to prepare high-foliar-affinity nanocapsules by coating the surfaces of abamectin-loaded PLA nanospheres with the coordination complexes of TA and Fe^3+^. In addition, hydrophobic cabbage and hydrophilic cucumber were used as two model crops to evaluate the target deposition of the Abam@PLA-TA nanocapsules. We expect to provide a universal surface modification method for improving the foliar deposition of pesticides through our work.

## 2. Materials and Methods

### 2.1. Materials

Abamectin (Abam, 95.6%) was purchased from Qilu Pharmaceutical Company, Ltd. (Hohhot, China). Poly(lactic acid) (PLA, M.W. approximately 100,000) was purchased from Daigang Biomaterial Company (Jinan, China). Poly(vinyl alcohol) (PVA, 87–90% hydrolyzed, average M.W. 30,000–70,000) was purchased from Sigma Aldrich (Darmstadt, Germany). Tannic acid (TA, 95%), iron chloride hexahydrate (FeCl_3_·6H_2_O, 99%), and rhodamine 6G (95%) were purchased from J&K chemical company (Beijing, China). The emulsifiable concentrate (EC) was provided by Noposion Agrochemicals Co., Ltd. (Shenzhen, China). Peach aphids (*Myzus persicae*) were supplied by the Pesticide Bioassay Lab at the Institute of Plant and Environment Protection, Beijing Academy of Agriculture and Forestry Sciences (Beijing, China). All other chemicals and reagents were used as received without further purification.

### 2.2. Preparation of Adhesive and Photostable Nanocapsules

**Figure 2:**
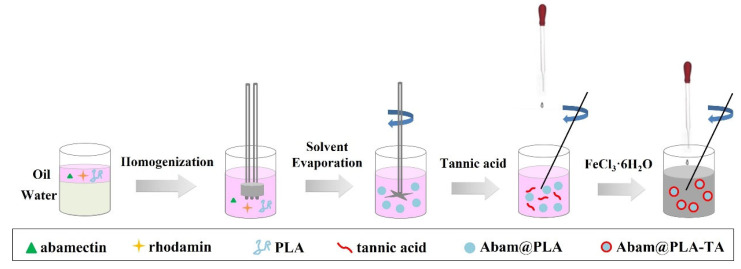
The technological process of Abam@PLA-TA nanocapsules.

The multi-functional nanocapsules were synthesized through the modification of tannic acid on the surface of abamectin-loaded nanospheres (Figure 2). First, the abamectin-loaded PLA nanospheres were prepared with the solvent evaporation method. Briefly, an oil in water (O/W) emulsion containing abamectin was stirred, the aqueous phase was prepared by dispersing 10 mg/mL PVA in 100 mL water, and the oil phase was obtained by dissolving 50 mg/mL abamectin and 50 mg/mL PLA in 25 mL dichloromethane. The oil phase was added dropwise into the aqueous phase by constantly stirring for 30 min to generate an O/W emulsion. Afterward, the as-prepared O/W emulsion was furtherly homogenized with the aid of an ultrasonic homogenizer (JY92, SCIENTZ, Ningbo, China). Then, the mixed emulsion was stirred vigorously (1000 rpm) at room temperature to completely evaporate the organic solvent. The resulting abamectin-loaded PLA nanospheres (denoted as Abam@PLA) were centrifuged and rinsed with an ethanol and water mixture (1:5, *v*/*v*) three times to remove the surfactant and abamectin on the surface of the nanospheres. Next, the collected pellet was redispersed in deionized water. Tannic acid (2.5 mL, 40 mg/mL) and iron chloride hexahydrate (2.5 mL, 10 mg/mL), in turn, were added to the Abam@PLA suspension, respectively. After stirring for 10 min, tannic acid was successfully coated onto the surface of Abam@PLA (denoted as Abam@PLA-TA). For the preparation of rhodamine-labeled Abam@PLA-TA nanocapsules, an extra 40 mg rhodamine 6G was added to the above oil phase.

### 2.3. Morphological and Structural Characterizations of the Nanocapsules

A scanning electron microscope (SEM; SU8010, Hitachi, Ibaraki Prefecture, Japan) was used to visualize the morphological characterizations of nanocapsules. The conductive resin was well covered with a little bit of the powder sample coated with gold. And then, the surface morphologies of the nanocapsules were observed at an accelerating voltage of 3 kV. In addition, the chemical elements on the surface of nanocapsules were determined with transmission electron microscopy (TEM, JEM-2010, JEOL LTD., Tokyo, Japan) after placing a drop of the suspension onto carbon-coated copper grids, and then electron-dispersive spectroscopy (EDS) was performed at an accelerating voltage of 200 kV. The hydrodynamic particle size and polydispersity index (PDI) of nanocapsules were investigated by dynamic light scattering (DLS, Zetasizer Nano-ZS90, Malvern, Worcestershire, UK). The drug loading rate (DLR) of Abam@PLA-TA nanocapsules was determined as follows: The Abam@PLA nanospheres (20 mg) were accurately weighed and extracted with 50 mL of a dichloromethane and methanol mixture (1:5, *v*/*v*) through sonification for 30 min at room temperature and later filtered with a 0.45 μm pore sizer. The concentration of abamectin was analyzed by a high-performance liquid chromatography system (HPLC, 1260 Infinity, Agilent, Santa Clara, CA, USA) equipped with a C18 column (5 mm, 4.6 × 150 mm, Agilent, USA) at 30 °C. The mobile phase was composed of a methanol and water mixture (9:1, *v*/*v*). The flow rate was 1 mL/min, and the UV detector wavelength was 245 nm. The Fourier transform infrared (FT-IR) spectra of the Abam@PLA nanospheres, tannic acid, and Abam@PLA-TA nanocapsules were recorded with an FT-IR spectrometer (FT-IR 6800, JASCO, Tokyo, Japan). All samples were prepared using potassium bromide pellets and measured over the spectral region between 4000 and 400 cm^−1^ at a resolution of 4 cm^−1^.

DLR (%) = (weight of abamectin encapsulated in nanoparticles/weight of nanoparticles) × 100.

Entrapment efficiency (EE, %) = (weight of abamectin encapsulated in nanoparticles/weight of feeding abamectin) × 100.

### 2.4. Performance Evaluation of the Nanocapsules

#### 2.4.1. In Vitro Release

Abam@PLA-TA nanocapsules (20 mg) were first suspended in 5 mL of 60% methanol solution and carefully transferred to a dialysis bag (2000 MWCO). Then, this bag was put into the release media (95 mL, 60% methanol solution) after being tightly sealed. The released abamectin was detected by collecting 2 mL of release media outside the dialysis bag after 1, 3, 6, 18, 31, 48, 72, 98, 120, 144, and 168 h. The abamectin concentration in the release media was detected with the abovementioned HPLC method. The accumulated release percentage was obtained over time. This release of Abam was completed at 25 °C and 100 rpm. At the same time, abamectin technical concentrate (TC) and Abam@PLA were measured for comparison.

#### 2.4.2. Retention Test on Live Foliage

The retention test was determined based on fluorescent images. All dust on the foliage surface was carefully removed to prevent any damage to the foliage. The rhodamine-labeled Abam@PLA-TA nanocapsule suspension (1 mL) was sprayed onto the surface of cucumber and cabbage leaves. After naturally drying in air, the fluorescence intensity was imaged at an excitation wavelength of 535 nm and detection wavelength of 580 nm (IVIS SPECTRUM, PerkinElmer, Shelton, CT, USA). Subsequently, each leaf was flushed with deionized water (100 mL), and the fluorescence intensity was recorded again. Each experiment was repeated three times, and the retention rate was calculated (RR). Moreover, the distributions of nanocapsules on the surfaces of the leaves were observed with SEM (3 kV, WD = 6.3 mm).

RR (%) = (the fluorescent intensity on the target leaf after flushing/the original fluorescent intensity on the same leaf) × 100.

#### 2.4.3. Stability Test

The Abam@PLA-TA suspension was dried in a glass dish at room temperature while being shielded from light. Then, these dishes were irradiated in a light incubator (XT5409-XPC80, Xutemp Technic Apparatus Co., Ltd., Hangzhou, China) at a distance of 10 cm and a temperature of 25 °C. A mercury lamp (100 W) equipped with a 365 nm light filter was applied as a UV radiation source, around which the test dishes could orbit. Then the dishes were collected at specific time intervals and the contents were dissolved in dichloromethane. The photodegradation behavior of the active ingredients after different irradiation times was analyzed by measuring the absorbance of each sample at 245 nm. At the same time, abamectin technical concentrate and Abam@PLA nanospheres were tested for comparison. In addition, the storage stability of Abam@PLA-TA nanocapsules was measured by dynamic light scattering and SEM after storage for 14 days at 0 °C, 25 °C, and 54 °C. All the treatments were administered three times. The photolysis of abamectin was characterized by first-order kinetic equation. The calculation formula is as follows:

*dC/dt* = *kC*

*C_t_* = *C*_0_*e*^−*kt*^

where *k* is the photolysis rate constant, h^−1^; *C*_0_ is the initial concentration of abamectin in the solution; and *C_t_* is the residual concentration at time *t*, mg/L. The half-life (*t*_1/2_) of degradation was calculated when *C_t_* was equal to *C*_0_/2.

### 2.5. Bioassays

The leaf dipping method was used to evaluate the indoor toxicity of the test samples, including Abam@PLA-TA, Abam@PLA, and commercial EC, towards peach aphids. Briefly, fresh cabbage leaves with a diameter of 6 cm were fully immersed in aqueous solutions of three kinds of abamectin test samples with concentrations of 1.56, 3.125, 6.25, 12.5, 25, 50, 100, and 200 ppm for 10 s and placed in culture dishes after naturally drying in air, and then 20 similar active aphids were inoculated on each dish. These treated dishes were sealed with microporous plastic wrap and incubated in specific conditions (75%, 25 °C). The number of dead aphids was counted after 48 h. This procedure was conducted four times for each group. The regression equation and fifty-percent lethal concentration (LC_50_) were calculated using DPS v12.01 statistical software. Deionized water was also tested for a blank comparison.

## 3. Results and Discussion

### 3.1. Characterization of Abam@PLA-TA Nanocapsules

**Figure 3:**
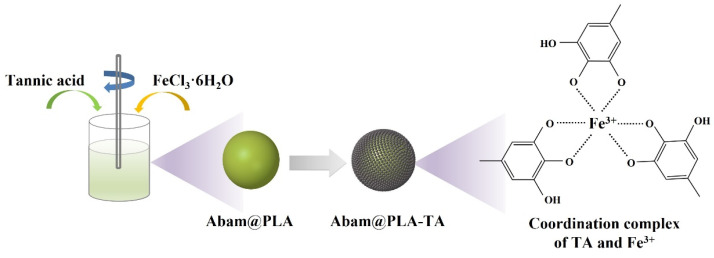
Schematic illustration of Abam@PLA-TA nanocapsules.

It is recognized that the metal chelating ability of polyphenols is related to the presence of ortho-dihydroxy polyphenols, i.e., molecules bearing catechol or galloyl groups [42,43]. Tannic acid consists of polygalloyl glucose molecules with different degrees of esterification, and the three galloyl groups of tannic acid can react with a metal ion to form a stable octahedral complex [44]. In this work, the natural polyphenol tannic acid and Fe^3+^ were chosen as the organic ligand and the inorganic cross-linker, respectively. A schematic illustration of the Abam@PLA-TA nanocapsules is shown in Figure 3. In the coating process, tannic acid was first deposited onto the surface of Abam@PLA nanospheres, and then Fe^3+^ triggered a coordination reaction with tannic acid to form a cross-linked film coating on the surface of the Abam@PLA nanosphere template.

**Figure 4:**
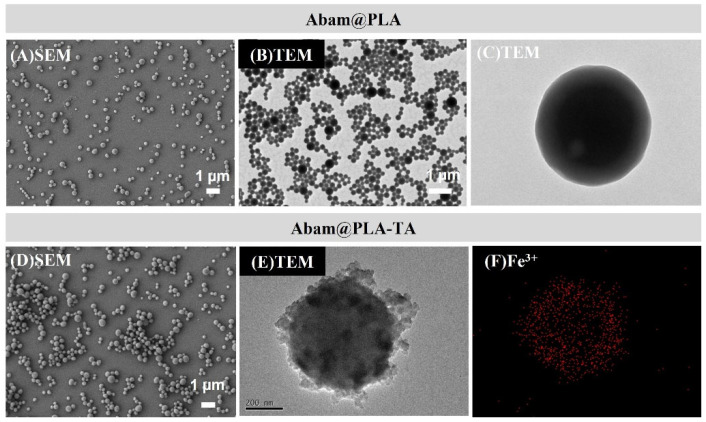
SEM, TEM, and EDS images of Abam@PLA and Abam@PLA-TA.

The morphology of abamectin-loaded nanoparticles was obtained using SEM and TEM (Figure 4). The results showed that Abam@PLA was a spherical particle with a smooth surface, but the surface of Abam@PLA-TA was rough due to a great number of TA-Fe^3+^ complexes generated from cross-linking between the phenolic hydroxyl groups of tannic acid and Fe^3+^ (Figure 4C,E). Moreover, a lot of Fe^3+^ ions were uniformly detected on the surface of Abam@PLA-TA, which indicated that the surfaces of the abamectin nanoparticles were coated with tannic acid (Figure 4F) [45,46]. The hydrodynamic size and drug loading rate of abamectin-loaded nanoparticles were consistent with the morphology results. The hydrodynamic size and drug loading rate of Abam@PLA were 451.8 nm and 46.5% (Figure S1). However, the size of Abam@PLA-TA was increased to 572.0 nm with the coating of the tannic acid-based wall, and the drug loading rate was 45.3% (Table 1).

**Figure 5:**
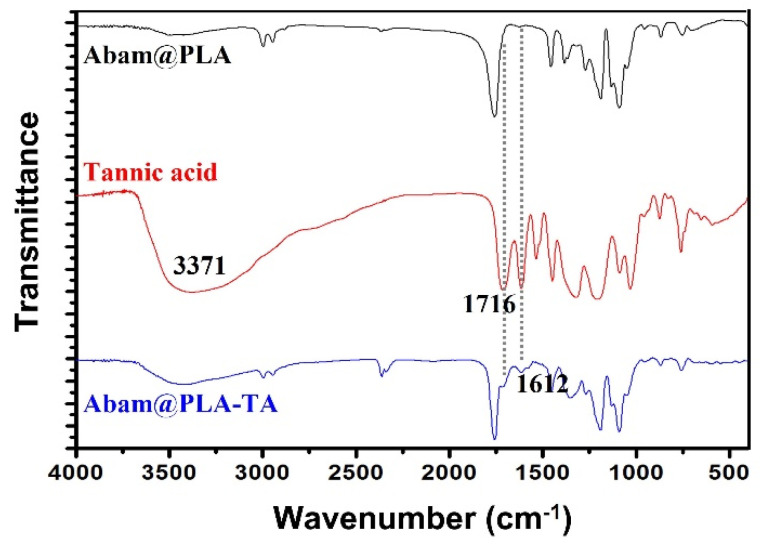
FTIR of Abam@PLA, tannic acid, and Abam@PLA-TA.

The FT-IR spectra of Abam@PLA-TA, Abam@PLA, and tannic acid are shown in Figure 5. For tannic acid, the prominent peak at 3371 cm^−1^ signifies the large number of phenols in the molecular structure, and the absorption peaks at 1716 and 1612 cm^−1^ were caused by the stretching vibrations of the C=O and cyclobenzene groups, respectively. Similarly, the above three characteristic peaks of tannic acid were also found in the spectra of Abam@PLA-TA nanocapsules, furtherly indicating that tannic acid was successfully coated onto the surface of the Abam@PLA nanospheres.

### 3.2. Sustained Release Behavior of Abam@PLA-TA Nanocapsules

**Figure 6:**
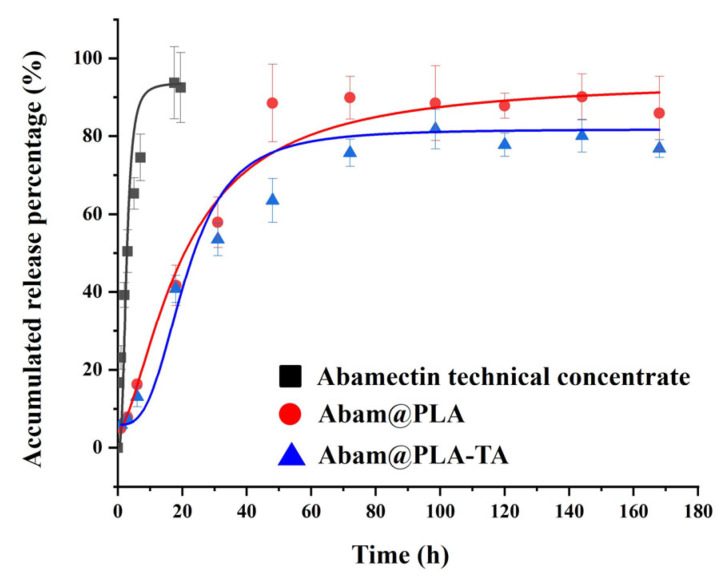
Release curves of abamectin technical concentrate, Abam@PLA, and Abam@PLA-TA.

The release curve of abamectin is shown in Figure 6. The release of the abamectin technical concentrate was rapid, with near-complete release achieved within 20 h. Its release rate was decreased significantly when loaded with PLA, indicating that PLA, as a pesticide carrier, is beneficial to the sustained release of the active ingredient and prolongs resistance to the target pest. Furthermore, the release rate of Abam@PLA-TA was slightly slower than that of Abam@PLA, implying that the tannic acid coating had a small effect on the release rate of abamectin. These results indicate that Abam@PLA-TA nanocapsules can act as an effective pesticide-loading system to regulate the sustained release of an active ingredient.

### 3.3. Stability of Abam@PLA-TA Nanocapsules

**Figure 7:**
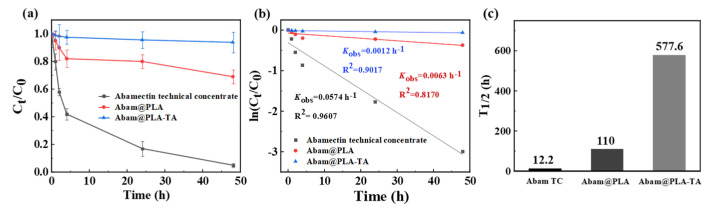
Photodegradation of abamectin technical concentrate, Abam@PLA, and Abam@PLA-TA versus irradiated time. The residual rate (**a**), fitted curves (**b**), and half-time (**c**) of abamectin technical concentrate, Abam@PLA, and Abam@PLA-TA.

The use of pesticides can easily be affected by various environmental factors, and sunlight is the most significant problem. The photodegradation rate of pesticides is related to their effective utilization and environmental safety. Microencapsulation enhances the photostability of light-sensitive pesticides, including azadirachtin, pyrethrin, trifluralin, and indoxacarb [47,48,49,50]. Abamectin is unstable under UV irradiation [51]. The protection of abamectin against photodegradation using a tannic acid–Fe^3+^ film as a wall coating material has not been fully investigated. In this work, tannic acid was used as a carrier and UV absorber of abamectin. The time-dependent photodegradation rate of abamectin technical concentrate, Abam@PLA, and Abam@PLA-TA is shown in Figure 7. The photolysis of abamectin can be characterized by a first-order kinetic equation. In the presence of a mercury lamp, the photodegradation of abamectin technical concentrate was very fast. The photolytic rate constant (*k*) for abamectin technical concentrate is 0.0574 h^−1^, while *k* is 0.0063 h^−1^ and 0.0012 h^−1^ for Abam@PLA and Abam@PLA-TA. The half-lives (*t*_1/2_) of photodegradation for abamectin TC, Abam@PLA, and Abam@PLA-TA are 12.1, 110.0, and 577.6 h, respectively, which clearly indicate that Abam@PLA-TA nanoparticles have better photostability than Abam@PLA because of their strong absorption derived from the polyphenol groups of tannic acid at wavelengths of 270–320 nm [29,52].

**Figure 8:**
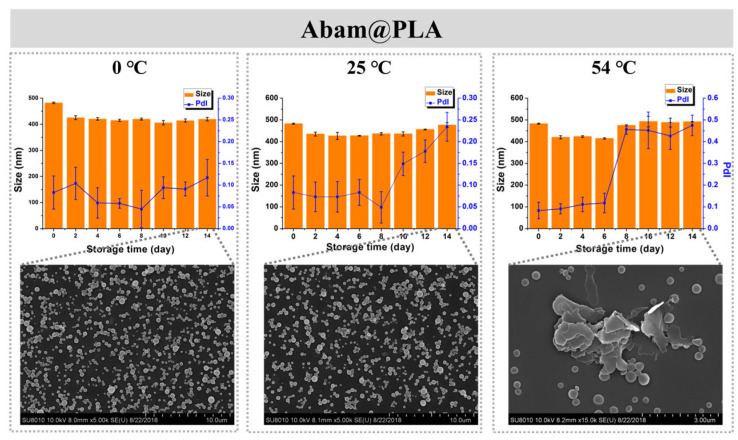
The time-dependent variation in size, PdI, and morphology of Abam@PLA at different temperatures.

**Figure 9:**
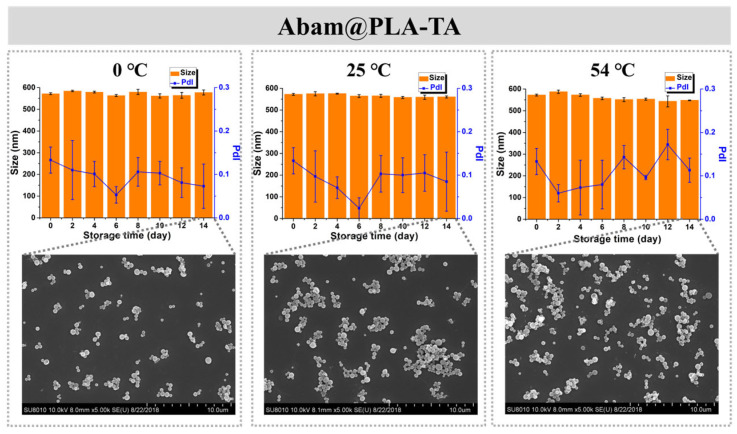
The time-dependent variation in size, PdI, and morphology of Abam@PLA-TA at different temperatures.

Storage stability is also an important index of a pesticide formulation. In this study, the storage stability of Abam@PLA and Abam@PLA-TA was investigated with SEM and DLS. As shown in Figure 8, the mean size and morphology of Abam@PLA nanospheres were stable at low (0 °C) and room (25 °C) temperatures, but the PdI increased significantly and large numbers of nanospheres were melted after 14 days of storage at a high temperature (54 °C), presumably because 54 °C is very close to the glass transition temperature of PLA. However, Abam@PLA-TA nanocapsules were very stable at the three test temperatures, indicating that the storage stability of Abam@PLA-TA nanocapsules can be enhanced by the tannic acid coating (Figure 9).

### 3.4. Foliar Affinity of Abam@PLA-TA Nanocapsules

**Figure 10:**
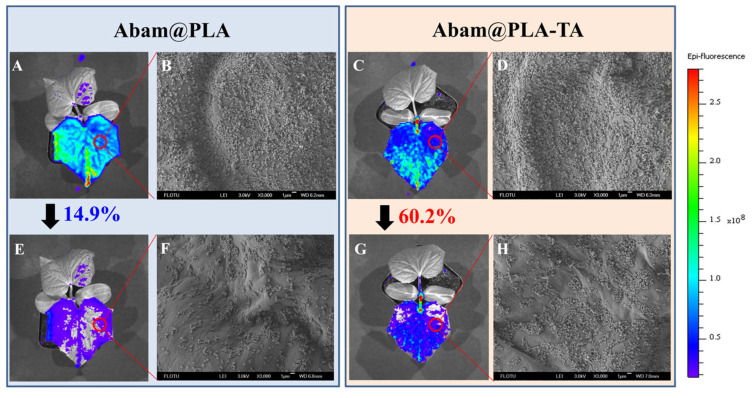
SEM and fluorescent images of cucumber leaves. (**A**–**D**) Images corresponding to Abam@PLA and Abam@PLA-TA, respectively. (**E**–**H**) Images corresponding to Abam@PLA and Abam@PLA-TA after washing, respectively.

**Figure 11:**
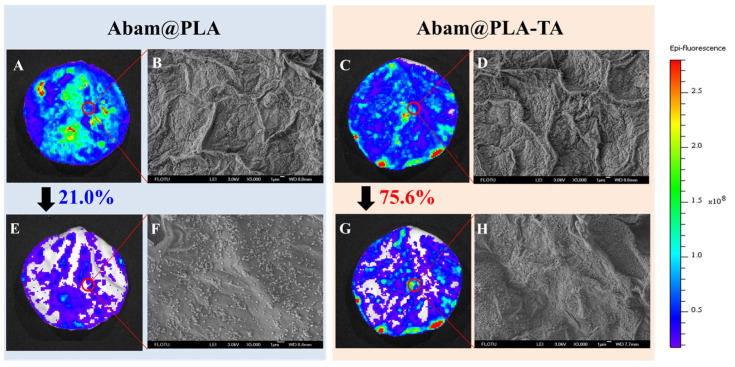
SEM and fluorescent images of cabbage leaves. (**A**–**D**) Images corresponding to Abam@PLA and Abam@PLA-TA, respectively. (**E**–**H**) Images corresponding to Abam@PLA and Abam@PLA-TA after washing, respectively.

**Figure 12:**
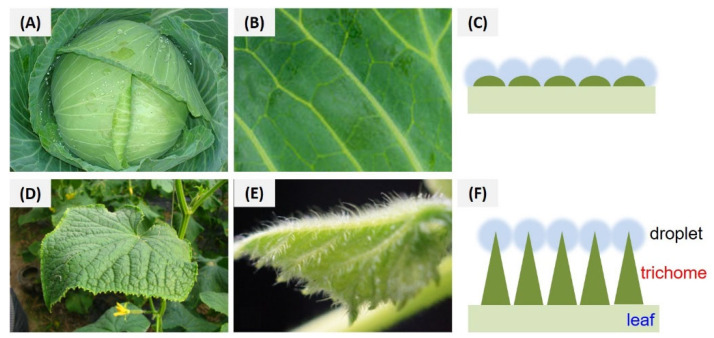
Morphological characteristics and deposition models of droplets on the trichomes of cabbage (**A**–**C**) and cucumber (**D**–**F**) leaves.

The efficient foliar deposition of pesticides is essential for improving the utilization of pesticides and minimizing the dosage. In general, it is difficult to visualize the deposition behavior of pesticides on an alive plant with conventional analytical methods. In this study, SEM and fluorescent imaging were used to research the foliar affinity of abamectin nanocapsules from both microscopic and macroscopic perspectives, respectively. As shown in the SEM images (Figure 10 and Figure 11), large numbers of nanospheres were dispersed irregularly on the surfaces of leaves, and many more Abam@PLA-TA nanocapsules remained on the leaves after flushing with deionized water 100 times, indicating that the surface modification of tannic acid improved the foliar affinity of Abam@PLA-TA nanocapsules. To accurately calculate the pesticide retention, fluorescent imaging was used to investigate the deposition of abamectin nanoparticles, which has the advantages of being both sensitive and convenient (Figure 10, Figure 11 and Figure S2). At the same time, the precision of this fluorescent imaging has been identified in previous studies [53,54]. In this experiment, rhodamine 6G, a type of fluorochrome that can mitigate the spontaneous fluorescence of chlorophyll, was encapsulated within these abamectin nanoparticles. The properties of fluorescent Abam@PLA-TA nanocapsules were the same as those of Abam@PLA-TA because less than 0.2% rhodamine 6G was included in the fluorescent nanoparticles. As clearly illustrated in the fluorescent images, the foliar retention of the nanoparticles was improved by the tannic acid coating. The retention rate of Abam@PLA and Abam@PLA-TA on cucumber leaves increased from 14.9% to 60.2%, and the deposition on cabbage leaves increased from 21.0% to 75.6% (Table S1). However, it is very interesting that the retention of Abam@PLA-TA on hydrophobic cabbage leaves was slightly higher than that of hydrophilic cucumber leaves without any help from surfactants. This may be due to the large number of trichomes covered on the surface of the cucumber foliage. These hair-like structures were both dense and soft, forming a physical barrier to prevent the droplets from coming into contact with the cucumber leaves (Figure 12). On the contrary, the cabbage leaves were smooth and glabrous, resulting in an enormous variety of bonds with tannic acid. These results confirmed that tannic acid definitely has super adhesion and can be used to enhance the foliar affinity of pesticides.

**Figure 13:**
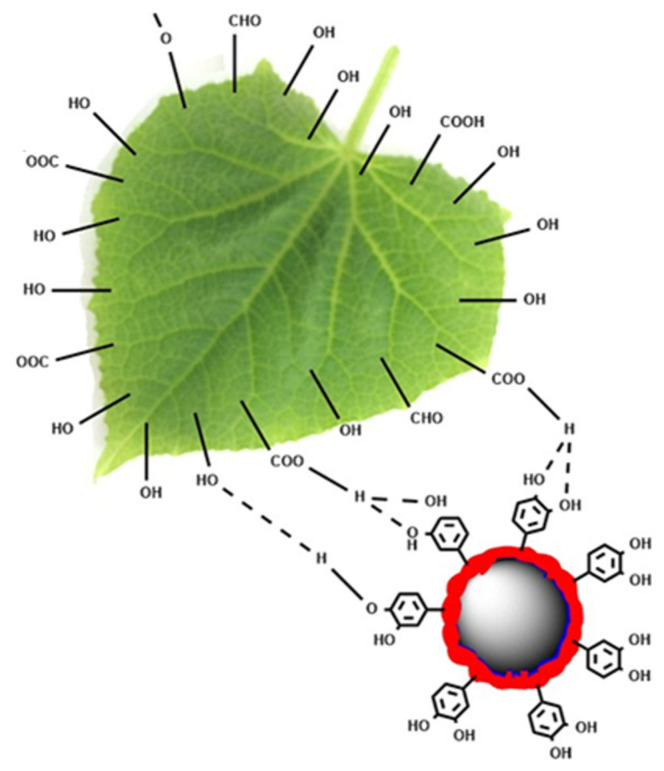
The chemical interactions between Abam@PLA-TA nanocapsules and the polar groups of foliar wax.

For most plants, the surface of a leaf is usually covered with a hydrophobic wax layer that comprises aliphatic and cyclic compounds, such as fatty acids, long-chain alkanes, primary and secondary alcohols, aldehydes, and ketones. These polar groups can react with tannic acid to form large numbers of hydrogen bonds [55,56]. In addition, coordination bonds may form between Abam@PLA-TA nanocapsules and crop foliage, owing to the ability of polyphenols to chelate various metal ions [42,43,57]. These multimodal bindings between Abam@PLA-TA nanocapsules and the foliage surfaces result in a strong affinity between them (Figure 13).

### 3.5. Biological Activity

**Figure 14:**
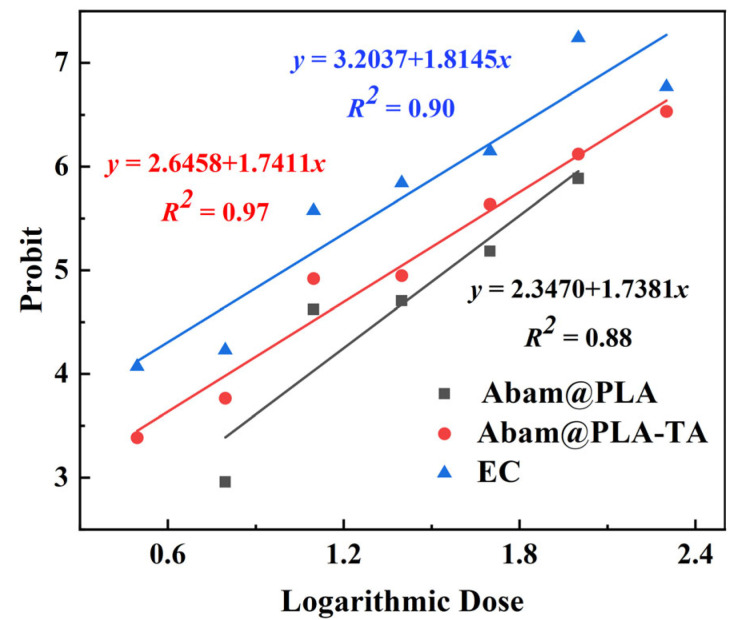
Full dose–response curves of three abamectin formulations against *Myzus persicae*.

The indoor toxicities of three abamectin formulations against aphids are summarized in Table 2 and Figure 14. Because the accumulated release percentages of Abam@PLA and Abam@PLA-TA were both less than 50% at 48 h, the acute toxicities of abamectin nanoparticles were lower than that of EC. However, the biological activity of Abam@PLA-TA was higher than that of Abam@PLA, indicating that the tannic acid coating enhanced the bioactivity of Abam@PLA-TA by increasing pesticide retention on the surface of hydrophobic cabbage leaves and enlarging the contact area between aphids and abamectin. These results show that Abam@PLA-TA may be more efficient in the sustained control of target pests, particularly in the later duration of crop growth, which helps to illustrate this scientific method of Abam@PLA-TA application.

## 4. Conclusions

In summary, a multi-functional abamectin-loaded nanocapsule was prepared, whose surface was modified with tannic acid. This nanocapsule was an irregular sphere with a diameter of around 572 nm. The Abam@PLA-TA nanocapsule exhibited better photostability and foliar retention than abamectin technical concentrate, which helps to provide better protection for target crops. The deposition of Abam@PLA-TA onto the surface of cucumber and cabbage leaves was investigated by fluorescent imaging and SEM. The adhesive properties of Abam@PLA-TA nanocapsules are highly dependent on their surface functional groups and can be markedly enhanced by tannic acid coating. The catechol groups of Abam@PLA-TA interacted with the polar groups of the wax layer, resulting in an increased retention rate from 14.9% to 75.6%. These results reveal a novel route to develop smart pesticide-loaded nanoparticles with foliar affinity, which helps to increase utilization efficiency, indirectly reducing the pesticide dosage and residue in the environment. At present, these conclusions are only applicable within the laboratory. In the future, field verification under real rainfall and with tank-mix adjuvants is very necessary.

## Figures and Tables

**Table 1:** Size, PDI, and DLR of Abam@PLA and Abam@PLA-TA.

	Content of TA (%)	Size (nm)	PDI	Drug Loading Rate (%)	Entrapment Efficiency (%)
Abam@PLA	-	451.8 ± 2.6	0.20 ± 0.03	46.5 ± 3.4	93.0 ± 0.1
Abam@PLA-TA	5.0	572.0 ± 4.4	0.30 ± 0.07	45.3 ± 3.0	90.6 ± 0.1

**Table 2:** Bioassay results of three abamectin formulations against *Myzus persicae* at 48 h.

Samples	*N*	Concentration (mg/L)	LC_50_ (95% CIs) (ppm)	Fit of Probit Line			
				Slope ± SE	*x* ^2^	df	*p*
Abam@PLA	600	1.5625–100	33.60 (26.78–44.01)	1.74 ± 0.23	7.20	3	0.07
Abam@PLA-TA	600	3.1250–200	22.50 (18.99–26.54)	1.74 ± 0.12	10.42	5	0.06
EC	600	3.1250–200	9.77 (5.03–14.76)	1.81 ± 0.15	22.84	5	0.01

## Data Availability

The data supporting the findings of this study are available within the article. Additional raw data are available from the corresponding author upon reasonable request.

## Supplementary Materials

The following supporting information can be downloaded at https://www.mdpi.com/article/10.3390/nano15231775/s1. Figure S1: Triplicate size-intensity curves of Abam@PLA nanoparticles; Figure S2: Radiant images of control leaves (excitation at 535 nm and detection at 580 nm); Table S1: Radiant efficiencies of control leaves and nanoparticles.
