# Supplementary material for: Surface Polyphenol Coordination Drives Efficient Foliar Deposition of Pesticide Nanocarriers
Source: Nanomaterials (Basel). 2025 Nov 26;15(23):1775. doi: 10.3390/nano15231775 (PMC12693096; doi:10.3390/nano15231775)
Supplement: Supplementary file 1 [file nanomaterials-15-01775-s001.zip › nanomaterials-3982612-supplementary.pdf]

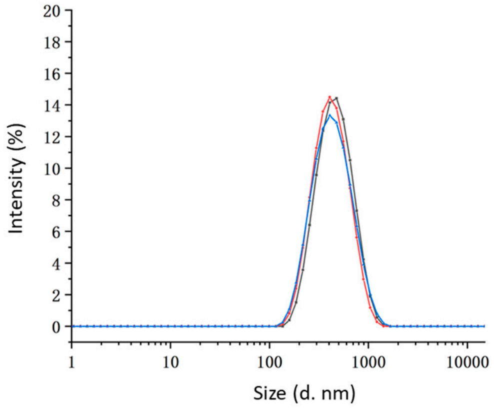


Figure S1. Triplicate size-intensity curves of Abam@PLA nanoparticles.

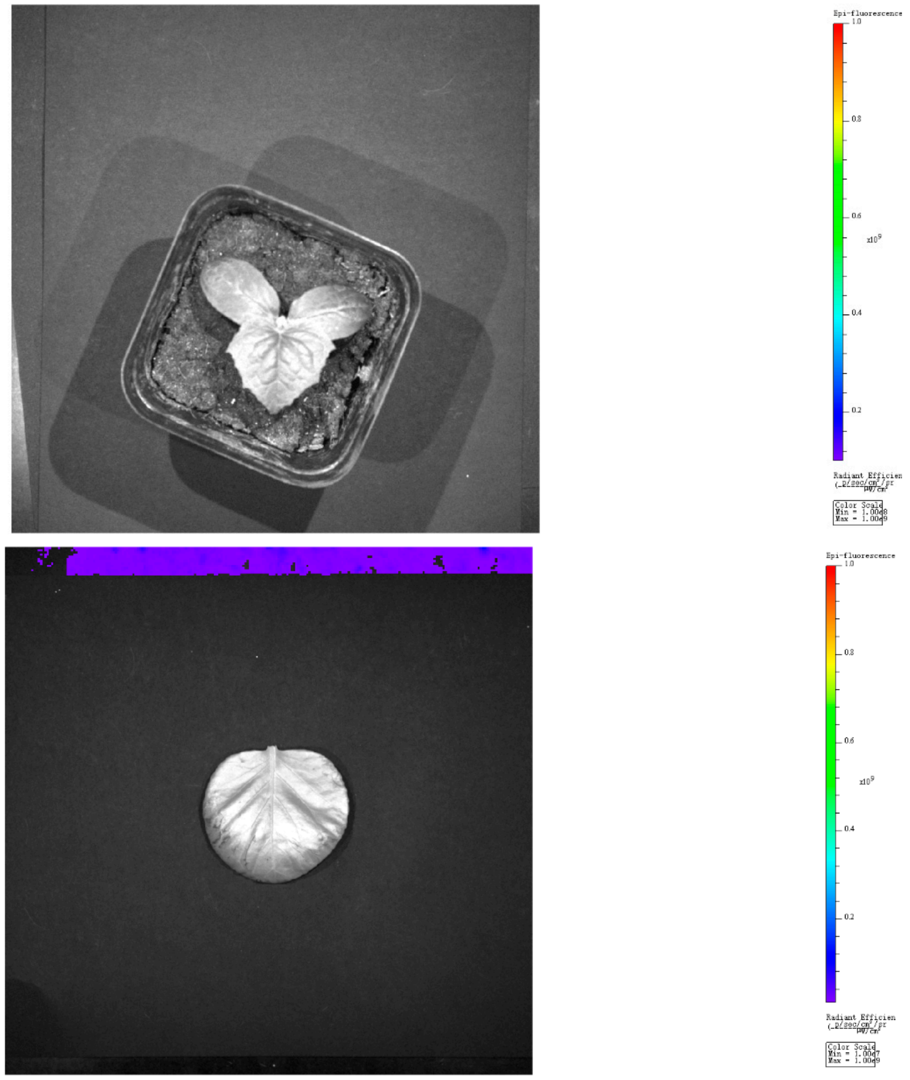


Figure S2. Radiant images of control leaves (excitation at 535 nm and detection at 580 nm).

Table S1. Radiant efficiencies of control leaves and nanoparticles.

| Crop | Sample | Radiant efficience (p/sec/cm$^2$/sr)/(μw/cm$^2$) | Radiant efficience after flushing (p/sec/cm$^2$/sr)/(μw/cm$^2$) | Rention rate (%) |
|---|---|---|---|---|
| Cucumber | Control | $3.61e^6 \pm 1.34e^5$ | - | - |
| | Abam@PLA | $2.30e^8 \pm 5.73e^7$ | $3.43e^7 \pm 1.95e^7$ | 14.9 |
| | Abam@PLA-TA | $1.60e^8 \pm 2.58e^7$ | $9.64e^7 \pm 1.47e^7$ | 60.2 |
| Cabbage | Control | $3.93e^6 \pm 2.19e^5$ | - | - |
| | Abam@PLA | $1.63e^8 \pm 3.21e^7$ | $3.42e^7 \pm 1.39e^7$ | 21.0 |
| | Abam@PLA-TA | $1.27e^8 \pm 1.47e^7$ | $9.58e^7 \pm 1.06e^7$ | 75.6 |
